# Effects of Virtual Reality on the Limb Motor Function, Balance, Gait, and Daily Function of Patients with Stroke: Systematic Review

**DOI:** 10.3390/medicina59040813

**Published:** 2023-04-21

**Authors:** Bohan Zhang, Ka-Po Wong, Jing Qin

**Affiliations:** 1 Centre for Smart Health, School of Nursing, The Hong Kong Polytechnic University, Hong Kong; bohan.zhang@connect.polyu.hk (B.Z.); harry.qin@polyu.edu.hk (J.Q.); 2 Department of Applied Social Sciences, The Hong Kong Polytechnic University, Hong Kong

**Keywords:** virtual reality, stroke, systematic review, rehabilitation, limb motor function, gait

## Abstract

*Background and Objectives*: This systematic review aimed to clarify the effectiveness of virtual reality rehabilitation on physical outcomes for people with stroke. *Materials and Methods*: Articles were searched through PubMed, EMBASE, the Cochrane Library, the Physiotherapy Evidence Database, CINAHL, Web of Science, and ProQuest Dissertations and Theses, from inception to 30 April 2022. Methodological quality was scored using the Assessing the Methodological Quality of Systematic Reviews 2 tool. Each systematic review for the outcome of interest was assessed by two independent reviewers using the Grading of Recommendations Assessment, Development, and Evaluation system. *Results*: Twenty-six articles were selected. These studies evaluated the effectiveness of virtual reality on limb motor function, balance, gait, and daily function in patients with stroke. The findings suggested a beneficial effect of virtual reality; there was a “very low” to “moderate” quality of evidence for improved limb extremity function, balance, and daily function, and a “very low” to “moderate” quality of evidence for improved gait. *Conclusions*: Despite widespread interest in the use of virtual reality rehabilitation, high-quality evidence for its routine use in stroke treatment is lacking. Further research is needed to determine the treatment modality, duration, and long-term effects of virtual reality on stroke populations.

## 1. Introduction

Stroke is the second leading cause of disability and death worldwide. In 2019, 12.2 million stroke events were reported, and the prevalence of stroke was 101 million [1]. Stroke is the main cause of cognitive deficits [2], and most stroke survivors suffer from long-term functional impairment. Current evidence suggests that most patients with cerebrovascular diseases with upper or lower limb injuries have persistent difficulties in dealing with the challenges of daily life, for instance, falls due to gait and balance problems [3]. Damage to the cerebral cortex affects patients’ physical function and motion ability and their quality of life [4].

Rehabilitation training can effectively improve the limb activity function of stroke patients, and reduce the rate of disability [5]. Traditional rehabilitation therapy relies heavily on physiotherapy and occupational therapy. Repetitive and task-specific exercises lead to controversy regarding the compliance and cost-effectiveness of traditional rehabilitation therapy, and its space and time constraints; the rehabilitation effect is highly related to the skills of the physical therapists, and traditional rehabilitation therapy cannot fully meet the needs of patients [6,7].

Virtual reality (VR) is achieved through computer hardware and software, whereby interactive simulations created by a computer provide participants with virtual environments similar to actual objects and events [8]. VR has been introduced as a potential new therapeutic approach to stroke rehabilitation and an alternative to physiotherapy and occupational therapy, which demonstrate only a modest effect on restoring motor function [8]. Choi et al. [9] found that post-stroke patients who used VR for upper extremity rehabilitation were satisfied with the procedure. Lloréns et al. [10] used VR for lower extremity rehabilitation in post-stroke patients, and showed that participants considered this approach to be highly usable.

Previous systematic reviews presented varied results regarding the effectiveness of VR. A meta-meta-analysis by Wu et al. [11] showed remarkable improvement in the recovery of upper limb function and balance in a VR group. However, the results showed considerable heterogeneity. Peng et al. [12] reported a substantial improvement in limb motor function among subacute stroke patients using VR for rehabilitation compared to conventional therapy. Compared to training without VR, Rooij et al. [13] found that VR training was more effective in improving balance or gait in stroke patients. In addition to these inconsistent results, the value of VR-related rehabilitation, such as effective methods, the timing of rehabilitation, and the intensity of rehabilitation, remains unclear.

The therapeutic value, including benefits and harms, associated with VR rehabilitation interventions for people with stroke must be determined. The most effective methods, timing, and intensity of these interventions warrant investigation. To our knowledge, no studies have comprehensively evaluated the existing systematic reviews of various rehabilitation interventions using VR. Therefore, this study aimed to systematically evaluate the evidence from systematic reviews of clinical trials; clarify the effectiveness of VR in the limb motor function, balance, gait, cognition, and daily function of patients with stroke; and explore the duration and form of rehabilitation using VR to provide a theoretical basis for clinical patient recovery.

## 2. Materials and Methods

This study was conducted following the Cochrane recommendations [14] and the Preferred Reporting Items for Overviews of SRs Including Harms (PRIO-harms) [15]. The protocol was prospectively registered on PROSPERO (CRD42022341986).

### 2.1. Search Methods

A systematic search of the following databases was conducted by two separate researchers (B.H. Zhang and K.P. Wong) from inception until 30 April 2022: PubMed, EMBASE, the Cochrane Library, the Physiotherapy Evidence Database (via the PEDro website), CINAHL, Web of Science, and ProQuest Dissertations and Theses. The following combinations of MeSH terms and free terms were used: “stroke”, “cerebrovascular disorders”, “virtual reality”, “computers”, and “systematic review”, and their synonyms. The reference lists of included studies were additionally reviewed. Disagreements were resolved through discussions among three researchers (B.H. Zhang, K.P. Wong, and J. Qin). Table 1 shows the research strategy for the PubMed database.

### 2.2. Eligibility Criteria

The inclusion and exclusion criteria of this review were established based on the PICOS principles, which facilitate the article selection process to enable the extraction of the most relevant studies. The inclusion criteria were as follows: patients with stroke aged over 18 years (P); VR rehabilitation therapy (I); conventional rehabilitation or placebo therapy (C); and outcome indicators that reflect the effectiveness of limb motor function, balance, gait, and daily function (O). Systematic reviews and/or meta-analyses of randomized controlled trials, cluster randomized controlled trials, and controlled clinical trials were included (S).

The exclusion criteria were as follows: (1) incomplete information (unable to obtain the required data, for example, where only an abstract was available, which would not enable retrieval of the full text, or where the outcomes of limb function, balance, gait, and daily function were not reported); (2) protocol, narrative reviews, and conference reviews; (3) duplicate records; and (4) non-English studies.

### 2.3. Study Selection and Data Extraction

After the duplicates were removed, the abstracts and titles of all studies were independently screened by the two researchers (B. Zhang and K.P. Wong), and the studies that did not meet the inclusion and exclusion criteria were excluded before further reading the full texts to determine final inclusion.

Separate Excel sheets were used by the two researchers (B. Zhang and K.P. Wong) to extract data. The information extracted from all reviews included the following: title, published year, published journal, first author, database, search terms, how many original studies were included and sample size, studies bias risk assessment methods, heterogeneity, intervention measures, data synthesis methods, and outcomes. The conflicts were discussed and resolved by three researchers (B. Zhang, K.P. Wong, and J. Qin).

### 2.4. Assessment of the Included Studies’ Methodological Quality

The methodologies included in this review were assessed using the Assessing the Methodological Quality of Systematic Reviews 2 (AMSTAR-2) tool [16]. AMSTAR 2 contains 16 items, among which items 2, 4, 7, 9, 11, 13, and 15 are the critical domains. If the answer to the item is correct and well-founded, then the judgment is “Yes”; if the answer to the item is correct but not well-founded, then the judgment is “partial Yes”; if the entry has no relevant evaluation information, then the judgment is “No”. Methodologies with no or one noncritical weakness(es) were rated “High”; those with more than one noncritical weakness were rated “Moderate”; those with one critical flaw with or without noncritical weaknesses were rated “Low”; and those with more than one critical flaw with or without noncritical weaknesses were rated as “Critically Low”.

In terms of quality of evidence, each systematic review of the outcomes of interest was assessed by two independent reviewers (B. Zhang and K.P. Wong) using the Grading of Recommendations Assessment, Development, and Evaluation (GRADE) system [17]. The GRADE system was based on five lower factors. The quality of evidence was rated “High”, “Moderate”, “Low”, or “Very Low”. The quality of evidence rating for randomized controlled trials (RCTs) was preset as “High”, downgraded by 1 to “Moderate”, downgraded by 2 to “Low”, and downgraded by 3 to “Very Low”. All disagreements were resolved by three researchers (B. Zhang, K.P. Wong, and J. Qin).

### 2.5. Data Synthesis

The characteristics of the included systematic reviews were described in a narrative manner. Differences in the participants, interventions, and types of data analysis in each review, and the main outcomes, were considered when assessing the effects of the interventions. Wherever possible, a summary of the results and of the statistical analyses for each included review is provided in summary tables and figures. More than one eligible review was found for VR and conventional rehabilitation. Common findings were reported when the reviews had similar conclusions, and the reasons for any differences related to the AMSTAR scores and the differences in participants, interventions, and type of data analysis included in the reviews were explored when the findings differed. Overlap in the trials included in the reviews that evaluated similar interventions was expected. In this case, the results were compared across all reviews and collapsed wherever possible.

## 3. Results

### 3.1. Search Results

**Figure 1:**
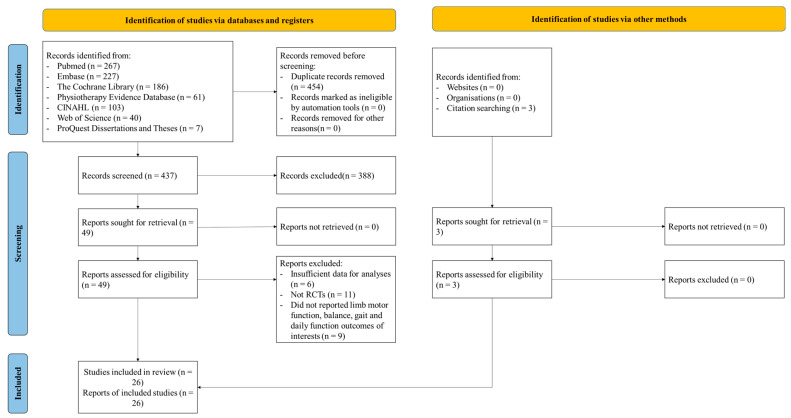
Flow diagram of literature screening.

A total of 891 studies were initially retrieved, and three reviews were determined by manually searching the references of related articles. After 454 duplicate articles were excluded, the title abstracts were read to exclude irrelevant literature. After further reading of the full texts, 26 systematic reviews were finally included. The PRISMA flow diagram of the study selection process is shown in Figure 1.

### 3.2. Study Characteristics

A total of 26 systematic reviews involving 22,031 adult participants with post-stroke disorders were included, and one of these reviews did not report the number of participants. This study identified 22 papers comprising systematic reviews and meta-analyses and 4 papers comprising systematic reviews only. The age range of participants was 18–94 years, and eight studies reported the sex of participants, with 5350 males and 3400 females.

Nine systematic reviews (n = 8740 participants) evaluated the effectiveness of VR on upper extremity functional recovery. Lower extremity functional recovery was reported in three systematic reviews (n = 1124), limb function (upper and lower extremity) was evaluated in five systematic reviews (n = 7255), the effectiveness of VR on balance function was reported in seven systematic reviews (n = 5356), gait was reported in five systematic reviews (n = 5019), and daily living skills were assessed in four systematic reviews (n = 5073).

Fourteen systematic reviews performed subgroup analyses regarding time since the onset of stroke, the intervention method, the type of VR, VR intervention duration, the frequency of intervention, control group type, the severity of paresis, outcomes, and study quality. The basic characteristics and a reference list of the included systematic reviews are shown in Table 2.

### 3.3. Quality of the Systematic Reviews

Among the 26 systematic reviews, 1 was rated “High”, 5 were rated “Moderate”, 14 were rated “Low”, and 6 were rated “Critically Low”. All 26 systematic reviews comprehensively searched the database, and seven of them also searched the grey databases and references in the included literature. All 26 systematic reviews reported the included studies’ basic characteristics and a list of excluded literature, and used appropriate bias tools to conduct a risk assessment. Among the 26 systematic reviews, 16 used The Physiotherapy Evidence Database scale, 7 used the Cochrane risk of bias tool, and 1 used The Downs and Black scale and the CONSORT checklist, the Jadad scale, and the ROBINS-2 tool. However, none of the 26 systematic reviews reported the reasons for the selection of RCTs and the funding of the original studies. Seven systematic reviews completed the registration of research methods ahead of schedule. Only one systematic review did not consider the effect of risk of bias on the results in its discussion. Given that four systematic reviews were only qualitative evaluations, no publication bias analysis was carried out. Among the remaining 22 meta-analyses, only seven analyzed publication bias. Table 3 demonstrates the results of the AMSTAR-2 quality evaluation.

The overall quality of the evidence for VR rehabilitation was assessed using the GRADE system (Table 4). Owing to the specific nature of this therapy, all included systematic reviews were at risk of bias in terms of blinding. Table 5 shows a synthesis of the best evidence on VR rehabilitation for patients with stroke.

### 3.4. Evidence Synthesis of VR Interventions

#### 3.4.1. Evidence Synthesis of Upper Limb Function

Fourteen systematic reviews assessed the outcome of VR in the rehabilitation of upper extremity motor function among patients with stroke. The results of 10 systematic reviews indicated that VR rehabilitation was more effective than traditional training in restoring upper limb function in stroke patients. Al-Whaibi et al. [22] and Laver et al. [8] suggested that VR rehabilitation training was effective but not statistically significant compared with conventional rehabilitation training. The study of Khan et al. [19] was divided into two parts: qualitative synthesis, which suggested that VR rehabilitation was effective, and meta-analysis, which showed that the comparison of Fugl-Meyer scores was not statistically significant.

#### 3.4.2. Evidence Synthesis of Lower Limb Function

Eight systematic reviews summarized the VR rehabilitation results for lower limb function in stroke patients. Corbetta et al. [38] indicated that patients with stroke demonstrated the effectiveness of limb function recovery only when they received VR intervention combined with conventional training. However, Laver et al. [8] found that the use of VR for rehabilitation in addition to usual nursing did not have a significant influence on patients’ motor function.

#### 3.4.3. Evidence Synthesis of Balance

Balance was reported in seven systematic reviews, all of which concluded that VR could provide better balance in stroke patients compared with conventional rehabilitation. After further analysis, VR rehabilitation was found to be more effective in the chronic phase in patients with stroke than in the acute phase (95% CI: 0.03–0.53, *p* = 0.03). Three studies found that the combination of traditional rehabilitation with VR could significantly improve the balance ability of patients, and is better than conventional rehabilitation alone. Iruthayarajah et al. [35] also observed that postural VR was better for balance function than other types of VR.

#### 3.4.4. Evidence Synthesis of Gait

Five studies reported gait, all of which confirmed that VR significantly improved walking speed and cadence in patients with stroke. Zhang et al. [20] found that a minimum of 5 weeks of VR intervention was needed for great improvements in gait and self-care in daily life (95% CI: 7.63–17.64, *p* < 0.001). Rooij et al. [13] stated that VR combined with conventional therapy and time-dose matching was more effective for training gait than conventional training (95% CI: 0.38–1.69, *p* = 0.002).

#### 3.4.5. Evidence Synthesis of Daily Function

Four systematic reviews reported the results of daily function. Only Aminov et al. [32] concluded that the rehabilitation effect of VR was consistent with that of conventional rehabilitation in terms of daily activities. Hence, their results were not considered significant. The remaining studies suggested that VR can better improve daily function than conventional rehabilitation.

#### 3.4.6. Evidence Synthesis of Subgroup Analysis

Through subgroup analysis, Laver et al. [8] pointed out that VR plus traditional rehabilitation training was highly beneficial for upper limb recovery. The same results were obtained by Fang et al. [24] and Li et al. [23]. Mekbib et al. [25] indicated that VR rehabilitation was highly effective in improving upper limb function in patients with subacute stroke. However, Al-Whaibi et al. [22] found that VR intervention can effectively improve upper limb function in subacute stroke and in the chronic phase of stroke. Fang et al. [24] found that immersive VR devices were better for rehabilitation than non-immersive VR. In addition, the duration and dose of VR were reported. Two systematic reviews found that a minimum of 10 sessions should be received by patients to ensure that the treatment is useful [8,24]. Laver et al. [8] and Mekbib et al. [25] noted that in VR training with an intervention duration of more than 15 h, the intervention group showcased great improvements in upper limb dysfunction and activity limitation compared with the control group. Li et al. [23] revealed that VR sessions lasting longer than 45 min for less than 6 weeks are highly beneficial to structure/function. Lee et al. [29] found that VR rehabilitation required a minimum of 5 weeks to improve the daily abilities of patients.

The overall results of this systematic review of the use of VR in stroke patients suggest “Very Low” to “Moderate” evidence quality for improved upper extremity function after stroke; “Very Low” to “Moderate” evidence quality for improved lower extremity function after stroke; “Very Low” to “Moderate” evidence quality for improved balance after stroke; “Very Low” and “Moderate” evidence quality for improved gait after stroke; and “Very Low” to “Moderate” evidence quality for improved daily function after stroke.

## 4. Discussion

This systematic review is the first study to comprehensively evaluate systematic reviews of VR’s efficacy in stroke patients. Twenty-six systematic reviews (758 RCTs with 22,031 participants) were included to summarize the best and latest evidence of the effectiveness of stroke rehabilitation interventions. This systematic approach to assessing review outcomes allows us to conduct a comparison of results from multiple reviews, providing a comprehensive evidence-based summary of the results. Our findings suggest beneficial effects of VR in improving limb function, balance, gait, and daily function, but the quality of evidence is low.

VR provides real-time multisensory feedback such as visual, auditory, and haptic feedback [42], and tracks patient performance and training details, such as the type and intensity of exercise [43]. The characteristics of VR make most RCTs unsuccessful in blinding participants and conductors, resulting in low-quality evidence. In addition, the systematic reviews included in this study involved different ethnicities and regions, resulting in high heterogeneity and the risk of inconsistent bias in quality assessment, which is one of the reasons for the low-quality evidence.

This study found that VR can successfully enhance the upper and lower limb function, balance, gait, and daily function of patients with stroke; however, high-quality evidence is severely lacking. VR positively affected functional recovery processes in patients with stroke, including pain reduction, muscle strengthening, and sensation recovery [29]. Lee et al. [44] used VR to intervene in the balance function of patients with stroke, and found that VR games had a positive effect on the balance of patients with stroke, who experienced greater pleasure during the intervention than during the standard treatment. Furthermore, VR improves the neural plasticity of patients with stroke by allowing them to perform functional task-specific activities in an enriched environment [5,45]. The high task variability, flexibility, and specificity of VR successfully boost patients’ motivation to comply with the therapeutic training [46]. Intensive therapy, the use of games to complete rewarding therapy, stimulus learning, and constructive feedback between stimulus and response are four components that can work together to ensure success through VR therapy [47].

Exercise intensity is a key factor in meaningful training after a stroke. This study found that VR requires at least 5–8 weeks, and a total time of more than 15 h of rehabilitation to improve upper extremity function, gait cadence, and self-care in the lives of patients with stroke. Stroke patients take some time to adjust to VR programs, it is crucial that patients undergo at least 8 weeks of VR training for adaptability [22]. Meanwhile, the recovery of patients’ structure/function was more evident when the session duration of VR exceeded 45 min compared with conventional therapy. This finding was identical to the recommendations for rehabilitation outlined in the national clinical guidelines for stroke in the United Kingdom [48]. Patients who received 45 min of daily upper extremity VR rehabilitation after a stroke experienced significant improvements in upper extremity function [49]. Moore et al. [50] implemented high-intensity rehabilitation training for 45–60 min per day in hospitalized patients with stroke, and found that the patients’ lower limb function and balance ability were significantly improved. However, none of the studies we included performed subgroup analyses of intervention frequency with VR effectiveness. By performing a pooled analysis of intervention frequency across all the included studies, we found 3–5 interventions per week to be an appropriate frequency. Further studies are needed in the future to validate this finding.

Using immersive VR technology, Mekbib et al. [51] found effective recovery of active motor function in patients with stroke. Since fully immersive VR brings participants into a 360° VR environment via a stereoscopic head-tracking head-mounted display, it provides effective treatment for impaired patients by enhancing the realism of experiencing another world [52]. This may be the reason why immersive VR is more effective than non-immersive VR.

This study was performed strictly in accordance with the PRISMA guidelines; however, some limitations may have influenced the results. First, although this study conducted a comprehensive search, only published studies were included, which may have led to selection bias. Second, due to the incompleteness of the included studies, this study did not summarize and analyze the follow-up data or the incidence of adverse effects of VR rehabilitation. Hence, the continuous effect and safety of VR rehabilitation cannot be determined. Given that this study focused on stroke patients’ limb motor function, balance, gait, and daily function outcomes, it did not include and analyze the effects of VR on the cognitive domain. With the increasing number of systematic reviews being published, an overlap in RCT data in the included systematic reviews may occur, leading to bias due to the inclusion of the same outcome data. However, according to the Cochrane Handbook [14], this overview presented and described the physical outcomes of stroke patients under VR intervention and summarized them without further data analysis, so the results are acceptable. Finally, although the selection and quality assessment of studies were carried out independently by two researchers with group consensus, the included studies were of low quality. Caution should be taken when interpreting the results of this systematic review.

## 5. Conclusions

With the development of technology, the role of VR rehabilitation in patients with stroke has received increasing awareness. Our review suggests that VR exercise for a duration of 5–8 weeks, with a session frequency of 3–5 days/week, for 45 min/day, and with a total time of more than 15 h can make this intervention very effective, although the quality of evidence that VR can effectively improve limb motor function, balance, gait, and daily function in patients with stroke is low. Owing to the unsatisfactory quality of the included studies and the lack of methodologically reliable trials, additional high-quality RCTs are needed in the future to prove the rehabilitation effects of VR and to further clarify its treatment modality, duration, and frequency for application as a complementary strategy for conventional rehabilitation.

## Figures and Tables

**Table 1:** Mapped medical subject headings (MeSH) terms and keywords employed in electronic search strategy.

Concept	MeSH Terms	Keywords
Stroke	Cerebrovascular disorders; basal ganglia cerebrovascular disease; brain ischemia; intracranial arterial diseases; intracranial arteriovenous malformations; intracranial embolism and thrombosis; intracranial hemorrhages; stroke; brain infarction; hemiplegia; paresis	Stroke; cva; poststroke; post-stroke; cerebrovasc*; hemipleg*; hemipar*; paresis; brain; cerebral*; cerebell*; brain*; vertebrobasilar
Virtual reality	User–computer interface; computers; microcomputers; computer systems; software; computer simulation; computer-assisted instruction; therapy, computer-assisted; computer graphics; video games	Virtual next reality*; virtual-reality; VR; video game*; video next gaming; gaming next console*; interactive next game; interactive next gaming; Nintendo next Wii; gaming next program*; haptics; haptic next device*
Systematic review	-	Systematic review; systematic overview; meta-analysis

* To search for all words with the same prefix.

**Table 2:** Characteristics of the included systematic reviews (n = 26).

Author, Year	Country	Included Articles	Participants	Intervention	Risk of Bias Assessment Tool	Focus	Meta-Analysis	Subgroup	Outcome	Main Findings
Angela Aguilera-Rubio, 2021 [18]	Spain	6	n = 144 Subacute stroke: 30 Chronic stroke: 65 Acute stroke: 8 Age range (years): 18–91	VR ^1^: the Leap Motion Controller system in VR environments CT ^2^ Frequency: 20 min/d~60 min/d; 3 d/w~5 d/w Duration: 2~12 weeks Number of sessions: 6~40 sessions	The Downs and Black scale and the CONSORT checklist	UL ^3^	No	No	Clinical scores: Upper Extremity Fugl-Meyer Assessment; Action Research Arm Test; Wolf Motor Function Test; Functional Independence Measure; and the Stroke Upper Limb Capacity Scale	After using VR, UL function in stroke patients was improved.
Azka Khan, 2021 [19]	Pakistan	150	n = 1617 Stroke Age range (years): 42–94 Time since stroke onset: 0.5 -> 12 months	VR: rehabilitation gaming system; electromagnetic motion tracker; IREX; Nintendo Wii Fit; RehabMasterTM system; RAPAEL Smart GloveTM; Kinect CT: physical therapy; occupational therapy Frequency: 5 min/d~120 min/d; 1 d/w~7 d/w Duration: 2~12 weeks Number of sessions: 9~56 sessions	The ROBINS-2 tool	Cognitive, UL, balance, LL ^4^	Yes	No	Clinical scores: Fugl-Meyer Assessment, Berg Balance scale; Mini Mental Scale Examination; Box and Block Test; 10 m Walk Test; Timed Up-and-Go test; The Manual Function Test; Action Research Arm	Qualitative synthesis: UL function and balance was improved in the VR group compared to non-VR group. Meta-analysis: For the MMSE score and the Fugl-Meyer score, the difference between the two groups was not statistically significant.
Bohan Zhang, 2021 [20]	China	87	n = 3540 Infarction stroke: 1664 Hemorrhage stroke: 866 Age range (years): 46–76 Time since stroke onset: 12.7 days–19.2 years Sex: male: 2000; female: 1329	VR: BioMster; STB-110; MOTOmed; GaitWatch; Xbox360; smart board; Leap Motion; Wii balance board; Kinect; Lokomat; IREX CT: physical therapy; occupational therapy; routine therapy Frequency: 15 min/d~100 min/d; 3 d/w~6 d/w Duration: 2~12 weeks Number of sessions: 6~40 sessions	The Cochrane risk of bias tool and the PEDro scale ^5^	UL, LL, balance, gait, cognition, and daily function	Yes	VR intervention duration	Clinical scores: Upper Extremity Fugl-Meyer Assessment; Action Research Arm Test; Wolf Motor Function Test; Box and Block Test; Lower Extremity Fugl-Meyer Assessment; Functional Ambulation Classification (FAC); Berg Balance Scale; 10 m Walk Test; Timed Up-and-Go Test; Velocity; Cadence; Mini Mental State Examination; Auditory Continuous Performance Test; Visual Continuous Performance Test; Functional Independence Measure; Modified Barthel Index	Stroke patients who received VR intervention showed considerable improvements in UL and LL movements, balance, walking, and self-care abilities.
Minxia Jin, 2021 [21]	China	40	n = 2018 Infarction stroke: 1311 Hemorrhage stroke: 433 Age range (years): 52–76 Time since stroke onset: 0.6–250 months Sex: male: 1252; female: 766	VR: Playstation EyeToy Games; RFVE; IREX; Xbox Kinect; BilMater; Nintendo Wii; RAPAEL Smart Glove CT: occupational therapy; usual activity; physical therapy Frequency: 20 min/d~60 min/d; 2 d/w~5 d/w Duration: 2~8 weeks Number of sessions: 10~30 sessions	The PEDro scale	UL, daily function	Yes	Severity of paresis; chronicity; type of control; type of virtual reality intervention; and degree of immersion	Clinical scores: Action Research Arm Test; Box and Block Test; Barthel Index; Brunnstrom stage; European Quality of Life 5-Dimension 5-Level Questionnaire; Functional Independence Measure; Fugl-Meyer Assessment Upper Extremity subscale; Jebsen Taylor Hand Function Test; Motor Activity Log—Amount of Use; Motor Activity Log—Quality of Movement; modified Ashworth Scale; modified Barthel Index; Manual Function Test; Manual Muscle Test; Range of Movement; Stroke Impact Scale; Wolf Motor Function Test; 9-Hole Peg Test	Compared to the control group, VR showed better results for overall arm function, and activity limitation. For participation and activity limits (specific tasks), no significant improvements were observed. More progress after training for patients with moderate-to-severe arm palsy. Greater beneficial impact with immersive virtual reality.
Reem M. Al-Whaibi, 2021 [22]	Saudi Arabia	6	n = 174 Infarction stroke: 65 Hemorrhage stroke: 18 Age range (years): 51–71 Sex: male: 124; female: 50	VR: Cy-Wee Z game; video games CT: physical therapy; gym therapy; occupational therapy Frequency: 30 min/d~90 min/d; 3 d/w~4 d/w Duration: 1~12 weeks Number of sessions: 6~18 sessions	The Cochrane risk of bias tool	UL	Yes	No	Clinical scores: Fugl-Meyer Assessment for upper extremities; Wolf Motor Function Test; Intrinsic Motivation Inventory; Lawton Instrumental Activities of Daily Living; Stroke Impact Scale; Manual Function Test; Box and Block Test; Chedoke McMaster Arm and Hand Activity Inventory; Fatigue Severity Scale; Motor Activity Log; Reaching Performance Scale in Stroke; Motor Activity Log-Amount Scale	Patients with chronic stroke showed a significant improvement within the group after receiving VR treatment. VR interventions produced similar results to traditional rehabilitation.
Yi Li, 2021 [23]	China	31	n = 1299 Subacute stroke: 544 Chronic stroke: 707 Acute stroke: 24 Age range (years): 49–69 Time since stroke onset: 0.5–95 months Sex: male: 827; female: 472	VR: Wii sports games; Nintendo; Kinect CT: physical therapy; occupational therapy Frequency: 30 min/d~60 min/d; 1 d/w~7 d/w Duration: 2~12 weeks Number of sessions: 8~42 sessions	The Cochrane risk of bias tool	UL	Yes	Session time (≤45 min vs. >45 min); intervention duration (<6 weeks vs. ≥6 weeks); sample size (n ≤ 30 vs. n >3 0; n, total enrolled participants).	Clinical scores: Upper Extremity Fugl-Meyer Assessment; Stroke Impact Scale; strength; grip strength; Motricity Index; Box and Block Test; Action Research Arm Test; Wolf Motor Function Test; modified Barthel Index; Jebsen Hand Function Test; Functional Independence Measure; Barthel Index; Motor Activity Log—Quality of Movement	For UL motor function recovery, VR was more effective than time dose-matched CT, and even more effective when using a virtual environment or VR mixed with CT. In contrast to CT, no improvement was achieved in patient performance and participation in daily activities with VR (VR only or VR mixed with CT). VE ^6^, a type of VR, was clearly superior to CG ^7^ in terms of movement of the overlying limbs. Structural/functional recovery benefited more from VR when session duration exceeded 45 min. When intervention duration was less than 6 weeks, VR was found to be more beneficial for structural/functional recovery.
Zongwei Fang, 2021 [24]	China	21	n = 619 Stroke Age range (years): 45–76 Affected side (left): 260	VR CT Frequency: 20 min/d~60 min/d; 1 d/w~5 d/w Duration: 4~8 weeks Number of sessions: 8~30 sessions	The Cochrane risk of bias tool	UL, balance	Yes	Session time (≥18 sessions versus <18 sessions); VR type (Immersive versus Non-Immersive)	Clinical scores: Fugl-Meyer Assessment–Upper Extremity; Box and Block Test; Functional Independence Measure; Berg Balance Scale	Traditional rehabilitation with VR rehabilitation outperformed traditional rehabilitation in terms of UL flexibility. In terms of activities of daily living and balance, there were no major differences between VR and traditional rehabilitation. Immersive VR may lead to more improvement in UL motor function than non-immersive VR.
Destaw B. Mekbib, 2020 [25]	China	27	n = 1094 Subacute stroke: 12 studies, range: 0.43–5.7 months Chronic stroke: 14 studies, range: 6.11–51 months; Age range (years): 64.48	VR: “off-the shelf” commercial video gaming console; custom-built virtual environment CT Frequency: not reported Duration: not reported Number of sessions: not reported	The PEDro scale	UL	Yes	Subacute stage (within 6 months) versus chronic stage (more than 6 months); and total amount of intervention: <15 h of intervention versus ≥15 h of intervention	Clinical scores: Fugl-Meyer Assessment for Upper Extremities; Box and Block Test; Motor Activity Log	VR group showed statistically significant improvement in the recovery of UL, activity, and participation versus the control group. When the intervention time exceeded 15 h, the VR group showed a significant improvement in the recovery of UL function. Improvement in the recovery of UL dysfunction was evident in subacute stroke patients but not in chronic patients.
Pablo Domı’nguez-Te´llez, 2020 [26]	France	20	n = 874 Stroke Age range (years): 53–76	VR: immersive VR; Xbox Kinect; 3D immersive VR; mechatronic VR; Nintendo Wii; Smart Glove; Armeo Spring CT Frequency: 30 min/d~60 min/d; 2 d/w~6 d/w Duration: 2~12 weeks Number of sessions: 10~30 sessions	The PEDro scale	UL, daily function	Yes	No	Clinical scores: Fugl-Meyer Assessment for the Upper Extremities; Box and Block Test; modified Barthel Index; Functional Independence Measure	The VR intervention was found to be effective for UL motor function and quality of life.
Shashank Ghai, 2020 [27]	Canada	32	n = 809 Stroke Age range (years): 41–81 Time since stroke onset: 19 days–15.1 years Sex: male: 541; female: 268	VR CT Frequency: 20 min/d~60 min/d; 2 d/w~5 d/w Duration: 2~12 weeks Number of sessions: 8~40 sessions	The PEDro scale	Gait	Yes	Intervention method	Clinical scores: 3 min Walk Test; 6 min Walk Test; 10 m Walk Test; 30 s Sit-to-Stand test; Activity-Specific Balance Confidence Scale; Action Reach Arm Test; Berg Balance Scale; Brunel Balance Assessment; Beck depression Inventory; cadence; Chedoke–McMaster Stroke Assessment; Fugl–Meyer Assessment; Four Square Step Test; Functional Reach Test; gate speed; Hamilton Depression Rating Scale; Lateral Reach Test; modified Ashworth Scale; modified Motor Assessment; muscle strength; Tinetti Performance-Oriented Mobility Assessment; Relationship Change Scale; Rivermead Mobility Index; sitting balance test; stride length; System Usability Scale; Tardieu scale; Timed Up-and-Go test; Visual Analog Scale; Walking Ability Questionnaire	VR training was beneficial for cadence, stride length, and speed.
Túlio Brandão XAVIER-ROCHA, 2020 [28]	Brazil	8	-	VR: Xbox Kinect; virtual reality games CT: standard therapy; task-oriented therapy Frequency: 30 min/d~120 min/d; 2 d/w~5 d/w Duration: 4~8 weeks Number of sessions: 12~40 sessions	The PEDro scale and Higgins visual scale	UL, LL, balance, gait, and daily function	No	No	Clinical scores: Fugl-Meyer Assessment—Upper extremity; Brunnstrom Stage Recovery; Box and Block test; Functional Independence Measure; Berg balance scale; Activity-Specific Balance Confidence Scale; Stroke Impact Scale; Fugl-Meyer Lower Extremities Assessment; Timed Up-and- Go Test; Manual Muscle test; Active range of motion	VR was effective for restoring balance, UL, and LL in post-stroke patients.
Han Suk Lee, 2019 [29]	Korea	21	n = 562 Stroke Age range (years): 46–72 Time since stroke onset: 6–87 months	VR: Wii balance board system; Nintendo Wii; treadmill training based real-world video; Xbox Kinect CT: standard training; physical therapy Frequency: 30 min/d~180 min/d; 2 d/w~5 d/w Duration: 2~8 weeks Number of sessions: 8~40 sessions	The PEDro scale	UL, LL, and daily function	Yes	The effects on functional improvement	Clinical scores: Rivermead mobility index; modified Ashworth Scale; postural sway velocity—AP eyes open; postural sway velocity—ML eyes closed; Berg Balance Scale; Timed Up-and-Go test; anteroposterior postural sway velocity; mediolateral postural sway velocity; postural sway velocity moment; Fugl-Myer Assessment; Short-Form 36 Health Survey; Wolf Motor Function Test; Reach to Grasp Test; Functional Reach Test; 10 m walking velocity; Box and Block Test; Manual Function Test; Functional Independence Measure; six-minute Walk Test; Wolf Motor Function Test; functional ability; Stroke-Specific Quality of Life Test—Upper Limb	VR was most effective in improving muscle tension, next to muscle strength.
Roghayeh Mohammadi, 2019 [30]	Iran	14	n = 344 Subacute stroke: 40 Chronic stroke: 265 Age range (years): 52–65 Time since stroke onset: 15 days- >1 year Sex: male: 182; female: 162	VR: Wii Fit balance board; virtual walking program; BalPro; IREX; BCT VR; Bio Rescue; Xbox Kinect CT: traditional rehabilitation Frequency: 20 min/d~45 min/d; 2 d/w~5 d/w Duration: 2~6 weeks Number of sessions: 10~20 sessions	The PEDro scale	Balance	Yes	No	Clinical scores: Brunel Balance Assessment; Berg Balance Scale; Functional Reach Test; modified Motor Assessment Scale; Timed Up-and-Go test	In terms of balance, the improvement was even more pronounced with the combination of VR and traditional therapy.
Sinae Ahn, 2019 [31]	Korea	34	n = 1604 Hemiplegic stroke	VR: gaming-based VR; RFVE; RehabMaster intervention CT: occupational therapy; Traditional rehabilitation Frequency: 20 min/d~60 min/d; 2 d/w~7 d/w Duration: 2~6 weeks Number of sessions: 10~24 sessions	The Jadad scale.	UL	Yes	No	Clinical scores: Action Research Arm Test; Barthel Index; Chedoke Arm and Hand Activity Inventory; Functional Independence Measure; Fugl-Meyer Assessment; motor activity log; modified Barthel index; Mini Mental State Examination; manual muscle testing; Wolf Motor Function Test.	Stroke patients’ UL function and independent mobility were effectively restored with VR exercises.
Anna Aminov, 2018 [32]	Australia	31	n = 971 Subacute stroke: 266 Chronic stroke: 602 Age range (years): 48–74 Time since stroke onset: 2–428 weeks	VR: virtual environment; commercial gaming CT Frequency: 20 min/d~60 min/d; 1 d/w~5 d/w Duration: 3~12 weeks Number of sessions: 4~24 sessions	The PEDro scale	UL, cognitive, and daily function	Yes	Intervention type; simulation type; study quality; recovery stage; control group type; duration; frequency; dose; daily intensity; weekly intensity	Clinical scores: Auditory Continuous Performance Test; Action Research Arm Test; Ashworth scale, Backward Digit Span Test; Backward Visual Span Test; Barthel Index; Box and Block test; color of word in word-color test; Composite Spasticity Index; Fugl-Meyer Assessment; Fugl-Meyer Assessment—Upper Extremity; Forward Digit Span Test; Forward Visual Span Test; Jebsen Hand Function Test; modified Ashworth Scale; Motor Activity Log; Manual Function Test; Motricity Index; Toulouse–Pieron Test; Visual Continuous Performance Test; Visual Learning Test; Verbal Learning Test; Trail Making Test A; Tower of London Test; Quality of Movement; Reaching Performance Scale for Stroke; Wolf Motor Function Test; Wechsler Memory Scale Third Edition; color of word in word-color test	The overall effect that VR generated extended beyond the effects of traditional therapies. Patient participation outcomes were not dramatically helpful.
Vilma Ferreira, 2018 [33]	Brazil	11	n = 310 Subacute stroke: 137 Chronic stroke: 154 Acute stroke: 19	VR: real-world video; Wii Kinect; balance-challenging virtual reality exercise CT: walking training; physiotherapy Frequency: 20 min/d~60 min/d; 2 d/w~5 d/w Duration: 2~12 weeks Number of sessions: 12~20 sessions	The PEDro scale	Balance and mobility	Yes	No	Clinical scores: two-minute Walk Test; Timed Up-and-Go Test; Intrinsic Motivation Inventory; Functional Ambulation Category; Berg Balance Scale	With the application of VR, balance was improved. However, there was no change in mobility.
Laver KE, 2017 [8]	Australia	72	n = 2470 Stroke Age range (years): 46–75	VR: commercially available gaming consoles; Nintendo Wii; Microsoft Kinect; gaming consoles; GestureTek IREX; Armeo; CAREN system CT: activity retraining; global motor function training Frequency: 20 min/d~90 min/d; 2 d/w~5 d/w Duration: 5~12 weeks Number of sessions: 8~36 sessions	The Cochrane ‘risk of bias’ tool	UL, LLL balance, and daily function	Yes	Dose of intervention; Time since onset of stroke; Specialised or gaming; Severity of impairment	Clinical scores: Action Research Arm Test; Canadian Occupational Performance Measure; Stroke Impact Scale; modified Rankin Scale; EQ5D; Motor Activity Log Arm Function Test; Useful Field of View Test; Barthel Index; Timed Up-and-Go Test; Functional Independence Measure; Box and Block test; Tapper Test; Fugl-Meyer UE; Chedoke Arm and Hand Activity Inventory; hand grip strength	Results were not statistically significant for UL function. For daily function, the between-group comparisons showed differences when virtual reality was combined with usual care. VR had the same effect on gait speed and balance as traditional rehabilitation.
Emma Maureen Gibbons, 2016 [34]	Australia	22	n = 552 Acute/subacute stroke: 190 Chronic stroke: 362 Age range (years): 41–78	VR: Wii Fit balance training; VR treadmill training; Xbox Kinect CT: standard care; treadmill training; ergometer bicycle training Frequency: 20 min/d~60 min/d; 2 d/w~6 d/w Duration: 2~12 weeks Number of sessions: 9~30 sessions	The PEDro scale	LL	Yes	No	Clinical scores: Berg Balance Scale; Timed Up-and-Go Test; Functional Reach Test; Stroke Rehabilitation Assessment of Movement Measure; 6 min Walk Test; medial–lateral; 10 m Walk Test; Performance-Oriented Assessment of Mobility	In the chronic stroke population, the VR group was found to favor balance, gait speed, stride length, and step length.
Ilona J.M. de Rooij, 2016 [13]	Netherlands	21	n = 516 Stroke Age range (years): 46–66 Time since stroke onset: 13 days–12 years	VR: VR treadmill training; VR balance training; virtual object training CT: conventional therapy; PNF exercise program; ergometer bicycle training Frequency: not reported Duration: not reported Number of sessions: not reported	The PEDro scale	Gait speed	Yes	Time dose-matched and VR-added conventional therapy	Clinical scores: 10 m Walk Test; Activity-Specific Balance Confidence; Berg Balance Scale; Functional Reach Test; medial–lateral, modified Motor Assessment Scale; Performance-Oriented Mobility Assessment; postural sway path length; postural sway velocity moment; Stability Index; Timed Up-and-Go Test; Walking Ability Questionnaire; Weight Distribution Index	VR training had a better effect on balance and gait recovery after stroke than traditional rehabilitation. For gait and balance, VR combined with conventional training provided better results than VR alone.
Jerome Iruthayarajah, 2016 [35]	Canada	20	n = 469 Infarction stroke: 127 Hemorrhage stroke: 101 Age range (years): 47–78 Time since stroke onset: 9.2–73.2 months Affected side (left): 156 Sex: male: 233; female: 203	VR: Xbox Kinect; Nintendo Wii Fit; treadmill walking; training with real-world video CT: ergometer bicycle training; task-oriented training; general exercise therapy Frequency: 30 min/d~60 min/d; 2 d/w~5 d/w Duration: 6~12 weeks Number of sessions: 10~40 sessions	The PEDro scale	Balance	Yes	Type of VR	Clinical scores: Berg Balance Scale; Timed Up-and-Go Test; Tinetti Performance-Oriented Mobility Assessment; Brunel Balance Assessment; 10 m Walking Test; Tinetti Performance-Oriented Mobility Assessment; Functional Reach Test	VR led to significant improvement in the balance of the patient.
Ling Chen, 2016 [36]	China	9	n = 265 Stroke Age range (years): 52–66 Time since stroke onset: 35 days–3 years	VR: IREX VR games; VR treadmill; Nintendo Wii Fit; IREX VR games CT: usual balance therapy; non-VR treadmill; physical therapy Frequency: 20 min/d~60 min/d; 3 d/w~5 d/w Duration: 3~5 weeks Number of sessions: 9~20 sessions	The PEDro scale	Balance	No	No	Clinical scores: Brunel Balance Assessment Category; Berg Balance Scale; Barthel Index; Balance Performance Monitoring; center of pressure; Functional Ambulation Categories; functional electrical stimulation; Fugl-Meyer Assessment; modified Motor Assessment Scale; Two-Minute Walk Test; Timed Up-and-Go Test	All but one study demonstrated positive improvements in static or dynamic balance.
Carlos Luque-Moreno, 2015 [37]	Spain	11	n = 231 Chronic stroke Age range (years): 47–66 Time since stroke onset: 1–11years	VR: Immersive VR; IREX VR system; Rutgers Ankle; WBB easy balance virtual rehabilitation CT Frequency: 20 min/d~60 min/d; 3 d/w~4 d/w Duration: 2~6 weeks Number of sessions: 6~20 sessions	The PEDro scale	LL	No	No	Clinical scores: 3 min Walk Test; 6 min Walk Test; 10 m Walk Test; 30 s Sit-to-Stand Test; Activity-Specific Balance Confidence Test; Anterior Reach Test; Brunel Balance Assessment; Berg Balance Scale; Functional Ambulatory Scale; Fugl-Meyer Scale; modified Motor Assessment Scale; Stepping Test; Timed Stair Test; Timed Up-and-Go Test	There was a significant improvement in gait speed, balance, and motor function following VR intervention. In more than 10 sessions, VR interventions had a positive impact on balance and gait. When combining VR with traditional physiotherapy, better results were obtained
Davide Corbetta, 2015 [38]	Italy	15	n = 341 Ischemic stroke Age range (years): 48–64 Sex: male: 191; female: 150	VR: Nintendo WBB; virtual outdoor environment; 3D virtual reality environment CT: treadmill walking training; conventional therapy Frequency: 20 min/d~60 min/d; 2 d/w~4 d/w Duration: 2~6 weeks Number of sessions: 6~20 sessions	The Cochrane Collaboration’s tool	LL, Balance, daily function	Yes	No	Clinical scores: 10 m Walk Test; 6 min Walk Test; Activity-Specific Balance Confidence Test; activities of daily living; Brunel Balance Assessment; Berg Balance Scale; Barthel Index; Community Walk Test; Functional Independence Measure; Fugl-Meyer Assessment; Functional Reach Test; Barthel Index; modified Motor Assessment Scale; Timed Up-and-Go Test; Functional Ambulatory Category	VR had benefits in terms of speed, balance, and mobility. Movement improved when VR was combined with formal rehabilitation training, but there were no significant advantages for walking speed and balance.
Zhen Li, 2015 [39]	China	16	n = 428 Acute/subacute stroke: 4 studies Chronic stroke: 12 Age range (years): 46–66	VR: Wii Fit VR; IREX VR; VR-based treadmill CT: treadmill; conventional therapy Frequency: 15 min/d~30 min/d; 2 d/w~5 d/w Duration: 3~12 weeks Number of sessions: 9~24 sessions	The Cochrane Collaborations ‘risk of bias’ tool	Balance	Yes	Time since stroke (less or more than six months), type of intervention	Clinical scores: Force Platform Indicators; Functional Reach Test; Activity-Specific Balance Confidence Scale; Berg Balance Scale; Timed Up-and-Go Test; Stability Index; functional electrical stimulation	People who received virtual reality interventions showed marked improvements in the Berg Balance Scale and the Timed Up and Go Test compared with controls. The difference between the “within six months of stroke” group and the “more than six months after stroke” group was not significant for balance. There were no significant differences between different intervention types for balance.
Juliana M. Rodrigues-Baroni, 2014 [40]	Brazil	7	n = 154 Chronic stroke Age range (years): 52–66 Time since stroke onset: 10–72 months	VR: virtual reality-based treadmill training; video game exercises CT: treadmill training; ankle movements Frequency: 20 min/d~60 min/d; 3 d/w~5 d/w Duration: 2~6 weeks Number of sessions: 6~20 sessions	The PEDro scale	Walking speed	Yes	No	Clinical scores: walking speed	Compared to the control group, the training with VR resulted in a significant increase in walking speed.
Keith R. Lohse, 2014 [41]	Canada	26	n = 626 Stroke Age range (years): 47–71 Time since stroke onset: 0.04–6.02 years	VR: 3D computer games; VR tasks; Wii balance board; IREX VR; VRBS training CT: standard occupation therapy Frequency: not reported Duration: not reported Number of sessions: not reported	The PEDro scale	UL, LL, Balance	Yes	VR type	Clinical scores: Action Research Arm Test; Brunel Balance Assessment; Berg Balance Scale; Box and Block Test; Functional Independence Measure; Fugl-Meyer Assessment; International Classification of Function, Disability, and Health; Jebsen–Taylor Hand Function Test; modified Barthel Index; Manual Function Test; modified Motor Assessment Scale; Postural Assessment Scale; Reaching Performance for Stroke Scale; Stroke Impact Scale; Timed Up-and-Go test; Wolf Motor Function Test; 10 m Walk Test	In terms of physical function and activity outcomes, compared to the traditional therapy group, VR therapy showed significant benefits.

^1^ VR: virtual reality; ^2^ CT: conventional therapy; ^3^ UL: upper limb; ^4^ LL: lower limb; ^5^ PEDro scale: The Physiotherapy Evidence Database Scale; ^6^ VE: virtual environments; ^7^ CG: commercially available gaming systems.

**Table 3:** Results of the Assessing the Methodological Quality of Systematic Reviews 2 tool quality evaluation (n = 26).

Author, Year	1	2	3	4	5	6	7	8	9	10	11	12	13	14	15	16	Quality of Studies
Angela Aguilera-Rubio, 2021 [18]	Y ^1^	N ^3^	N	PY ^2^	Y	N	Y	Y	Y	N	NP ^4^	NP	Y	N	NP	Y	Low
Azka Khan, 2021 [19]	Y	PY	N	PY	Y	Y	Y	Y	Y	N	Y	Y	Y	N	N	Y	Low
Bohan Zhang, 2021 [20]	Y	Y	N	Y	Y	Y	Y	Y	Y	N	Y	Y	Y	Y	N	Y	Low
Minxia Jin, 2021 [21]	Y	Y	N	Y	Y	Y	Y	Y	Y	N	Y	Y	Y	Y	N	Y	Low
Reem M. Al-Whaibi, 2021 [22]	Y	PY	N	Y	Y	Y	Y	Y	Y	N	Y	Y	Y	N	Y	Y	High
Yi Li, 2021 [23]	Y	N	N	PY	N	Y	Y	Y	Y	N	Y	Y	Y	Y	N	Y	Low
Zongwei Fang, 2021 [24]	Y	Y	N	PY	N	Y	Y	Y	Y	N	Y	Y	Y	Y	Y	Y	Moderate
Destaw B. Mekbib, 2020 [25]	Y	N	N	PY	N	Y	Y	Y	Y	N	Y	Y	Y	N	N	Y	Moderate
Pablo Domı´nguez-Te´ llez, 2020 [26]	Y	PY	N	PY	Y	Y	Y	Y	Y	N	Y	Y	Y	N	N	Y	Low
Shashank Ghai, 2020 [27]	Y	Y	N	PY	Y	Y	Y	Y	Y	N	Y	Y	Y	Y	Y	Y	Moderate
Túlio Brandão XAVIER-ROCHA, 2020 [28]	Y	PY	N	Y	Y	N	Y	Y	Y	N	NP	NP	Y	N	NP	Y	Moderate
Han Suk Lee, 2019 [29]	Y	N	N	PY	Y	Y	Y	Y	Y	N	Y	Y	Y	N	Y	Y	Low
Roghayeh Mohammadi, 2019 [30]	Y	PY	N	PY	Y	Y	Y	Y	Y	N	Y	Y	Y	Y	N	N	Low
Sinae Ahn, 2019 [31]	Y	N	N	PY	Y	Y	Y	Y	Y	N	Y	Y	Y	Y	Y	Y	Low
Anna Aminov, 2018 [32]	Y	N	N	PY	Y	Y	Y	Y	Y	N	Y	Y	Y	N	N	Y	Critically Low
Vilma Ferreira, 2018 [33]	Y	Y	N	PY	Y	N	Y	Y	Y	N	Y	Y	Y	Y	Y	Y	Moderate
Laver KE, 2017 [8]	Y	N	N	Y	Y	Y	Y	Y	Y	N	Y	Y	Y	Y	Y	Y	Low
Emma Maureen Gibbons, 2016 [34]	Y	N	N	PY	Y	Y	Y	Y	Y	N	Y	Y	Y	N	N	N	Critically Low
Ilona J.M. de Rooij, 2016 [13]	Y	N	N	PY	Y	Y	Y	Y	Y	N	Y	Y	Y	Y	N	Y	Critically Low
Jerome Iruthayarajah, 2016 [35]	Y	PY	N	Y	Y	Y	Y	Y	Y	N	Y	Y	Y	N	N	N	Low
Ling Chen, 2016 [36]	Y	N	N	Y	Y	Y	Y	Y	Y	N	NP	NP	Y	N	NP	Y	Low
Carlos Luque-Moreno, 2015 [37]	Y	N	N	PY	N	N	Y	Y	Y	N	NP	NP	Y	N	NP	Y	Low
Davide Corbetta, 2015 [38]	Y	N	N	PY	Y	N	Y	Y	Y	N	Y	Y	Y	Y	N	Y	Critically Low
Zhen Li, 2015 [39]	Y	Y	N	PY	Y	Y	Y	Y	Y	N	Y	Y	N	N	N	Y	Critically Low
Juliana M. Rodrigues-Baroni, 2014 [40]	Y	N	N	PY	Y	Y	Y	Y	Y	N	Y	Y	Y	Y	N	Y	Critically Low
Keith R. Lohse, 2014 [41]	Y	Y	N	PY	Y	Y	Y	Y	Y	N	Y	Y	Y	Y	N	Y	Low

^1^ Y = Yes, ^2^ PY = partial Yes, ^3^ N = No, ^4^ NP = meta-analysis was not performed.

**Table 4:** Results of the Grading of Recommendations Assessment, Development, and Evaluation system (n = 26).

Author, Year	Lower Factors					
	Risk of Bias	Inconsistence	Indirectness	Imprecision	Publication Bias	Quality of Evidence (GRADE)
Angela Aguilera-Rubio, 2021 [18]	−1	0	0	−1	0	Low
Azka Khan, 2021 [19]	−1	−1	0	0	−1	Very Low
Bohan Zhang, 2021 [20]	−1	−1	0	0	−1	Very Low
Minxia Jin, 2021 [21]	−1	−1	0	0	−1	Moderate
Reem M. Al-Whaibi, 2021 [22]	−1	0	0	−1	0	Low
Yi Li, 2021 [23]	−1	0	0	0	−1	Low
Zongwei Fang, 2021 [24]	−1	0	0	0	0	Moderate
Destaw B. Mekbib, 2020 [25]	−1	−1	0	0	−1	Low
Pablo Domı´nguez-Te´ llez, 2020 [26]	−1	−1	0	0	−1	Very Low
Shashank Ghai, 2020 [27]	−1	0	0	0	0	Moderate
Túlio Brandão XAVIER-ROCHA, 2020 [28]	−1	0	0	0	0	Moderate
Han Suk Lee, 2019 [29]	−1	0	0	0	0	Moderate
Roghayeh Mohammadi, 2019 [30]	−1	0	0	−1	−1	Very Low
Sinae Ahn, 2019 [31]	−1	0	0	0	0	Moderate
Anna Aminov, 2018 [32]	−1	0	0	0	−1	Low
Vilma Ferreira, 2018 [33]	−1	0	0	−1	0	Moderate
Laver KE, 2017 [8]	−1	0	0	0	0	Moderate
Emma Maureen Gibbons, 2016 [34]	−1	−1	0	0	−1	Very Low
Ilona J.M. de Rooij, 2016 [13]	−1	−1	0	0	−1	Very Low
Jerome Iruthayarajah, 2016 [35]	−1	0	0	0	−1	Low
Ling Chen, 2016 [36]	−1	0	0	−1	0	Low
Carlos Luque-Moreno, 2015 [37]	−1	0	0	−1	0	Low
Davide Corbetta, 2015 [38]	−1	0	0	−1	−1	Very Low
Zhen Li, 2015 [39]	−1	0	0	0	−1	Low
Juliana M. Rodrigues-Baroni, 2014 [40]	−1	0	0	−1	−1	Very Low
Keith R. Lohse, 2014 [41]	−1	−1	0	0	−1	Very Low

**Table 5:** Synthesis of the best evidence (n = 26).

Evidence	Number of Studies and Participants	Number of GRADE ^1^ Results			
		Very Low	Low	Moderate	High
Upper limb function	554 RCTs 16,986 participants	3	5	6	0
Lower limb function	262 RCTs 7696 participants	4	1	3	0
Balance	165 RCTs 5356 participants	2	3	2	0
Gait	155 RCTs 5019 participants	3	0	2	0
Daily function	147 RCTs 5073 participants	1	1	2	0

^1^ GRADE: Grading of Recommendations Assessment, Development, and Evaluation.

## Data Availability

Not applicable.

## References

[1] GBD 2019 Stroke Collaborators (2021). Global, regional, and national burden of stroke and its risk factors, 1990–2019: A systematic analysis for the Global Burden of Disease Study 2019. Lancet Neurol..

[2] Zhao Y., Zhang X., Chen X., Wei Y. (2022). Neuronal injuries in cerebral infarction and ischemic stroke: From mechanisms to treatment (Review). Int. J. Mol. Med..

[3] Ochi M., Wada F., Saeki S., Hachisuka K. (2015). Gait training in subacute non-ambulatory stroke patients using a full weight-bearing gait-assistance robot: A prospective, randomized, open, blinded-endpoint trial. J. Neurol. Sci..

[4] Axer H., Axer M., Sauer H., Witte O.W., Hagemann G. (2010). Falls and gait disorders in geriatric neurology. Clin. Neurol. Neurosurg..

[5] Dąbrowski J., Czajka A., Zielińska-Turek J., Jaroszyński J., Furtak-Niczyporuk M., Mela A., Poniatowski Ł.A., Drop B., Dorobek M., Barcikowska-Kotowicz M. (2019). Brain functional reserve in the context of neuroplasticity after stroke. Neural Plast..

[6] Shen J., Gu X., Yao Y., Li L., Shi M., Li H., Sun Y., Bai H., Li Y., Fu J. (2022). Effects of virtual reality-based exercise on balance in patients with stroke: A systematic review and meta-analysis. Am. J. Phys. Med. Rehabil..

[7] Lesauskaitė V., Damulevičienė G., Knašienė J., Kazanavičius E., Liutkevičius A., Janavičiūtė A. (2019). Older adults-potential users of technologies. Medicina.

[8] Laver K.E., Lange B., George S., Deutsch J.E., Saposnik G., Crotty M. (2017). Virtual reality for stroke rehabilitation. Cochrane Database Syst. Rev..

[9] Choi Y.H., Paik N.J. (2018). Mobile Game-based Virtual Reality Program for Upper Extremity Stroke Rehabilitation. J. Vis. Exp..

[10] Lloréns R., Noé E., Colomer C., Alcañiz M. (2015). Effectiveness, usability, and cost-benefit of a virtual reality-based telerehabilitation program for balance recovery after stroke: A randomized controlled trial. Arch. Phys. Med. Rehabil..

[11] Wu J., Zeng A., Chen Z., Wei Y., Huang K., Chen J., Ren Z. (2021). Effects of virtual reality training on upper limb function and balance in stroke patients: Systematic review and meta-meta-analysis. J. Med. Internet Res..

[12] Peng Q.C., Yin L., Cao Y. (2021). Effectiveness of virtual reality in the rehabilitation of motor function of patients with subacute stroke: A meta-analysis. Front. Neurol..

[13] De Rooij I.J., van de Port I.G., Meijer J.G. (2016). Effect of virtual reality training on balance and gait ability in patients with stroke: Systematic review and meta-analysis. Phys. Ther..

[14] Pollock M., Fernandes R., Becker L., Pieper D., Hartling L., Higgins J.P.T., Thomas J., Chandler J., Cumpston M., Li T., Page M. (2021). Overviews of Reviews. Cochrane Handbook for Systematic Reviews of Interventions Version 6.2.

[15] Bougioukas K.I., Liakos A., Tsapas A., Ntzani E., Haidich A.B. (2018). Preferred reporting items for overviews of systematic reviews including harms checklist: A pilot tool to be used for balanced reporting of benefits and harms. J. Clin. Epidemiol..

[16] Shea B.J., Reeves B.C., Wells G., Thuku M., Hamel C., Moran J., Moher D., Tugwell P., Welch V., Kristjansson E. (2017). AMSTAR 2: A critical appraisal tool for systematic reviews that include randomised or non-randomised studies of healthcare interventions, or both. BMJ.

[17] Balshem H., Helfand M., Schünemann H.J., Oxman A.D., Kunz R., Brozek J., Vist G.E., Falck-Ytter Y., Meerpohl J., Norris S. (2011). GRADE guidelines: 3. Rating the quality of evidence. J. Clin. Epidemiol..

[18] Aguilera-Rubio Á., Alguacil-Diego I.M., Mallo-López A., Cuesta-Gómez A. (2022). Use of the Leap Motion Controller^®^ System in the rehabilitation of the upper limb in stroke: A systematic review. J. Stroke Cerebrovasc. Dis..

[19] Khan A., Podlasek A., Somaa F. (2021). Virtual reality in post-stroke neurorehabilitation—A systematic review and meta-analysis. Top. Stroke Rehabil..

[20] Zhang B., Li D., Liu Y., Wang J., Xiao Q. (2021). Virtual reality for limb motor function, balance, gait, cognition and daily function of stroke patients: A systematic review and meta-analysis. J. Adv. Nurs..

[21] Jin M., Pei J., Bai Z., Zhang J., He T., Xu X., Zhu F., Yu D., Zhang Z. (2022). Effects of virtual reality in improving upper extremity function after stroke: A systematic review and meta-analysis of randomized controlled trials. Clin. Rehabil..

[22] Al-Whaibi R.M., Al-Jadid M.S., ElSerougy H.R., Badawy W.M. (2021). Effectiveness of virtual reality-based rehabilitation versus conventional therapy on upper limb motor function of chronic stroke patients: A systematic review and meta-analysis of randomized controlled trials. Physiother. Theory Pract..

[23] Li Y., Huang J., Li X., Qiao J., Huang X., Yang L., Yu H. (2021). Effect of time-dose-matched virtual reality therapy on upper limb dysfunction in patients poststroke: A meta-analysis of randomized controlled trials. Arch. Phys. Med. Rehabil..

[24] Fang Z., Wu T., Lv M., Chen M., Zeng Z., Qian J., Chen W., Jiang S., Zhang J. (2022). Effect of traditional plus virtual reality rehabilitation on prognosis of stroke survivors: A systematic review and meta-analysis of randomized controlled trials. Am. J. Phys. Med. Rehabil..

[25] Mekbib D.B., Han J., Zhang L., Fang S., Jiang H., Zhu J., Roe A.W., Xu D. (2020). Virtual reality therapy for upper limb rehabilitation in patients with stroke: A meta-analysis of randomized clinical trials. Brain Inj..

[26] Domínguez-Téllez P., Moral-Muñoz J.A., Salazar A., Casado-Fernández E., Lucena-Antón D. (2020). Game-based virtual reality interventions to improve upper limb motor function and quality of life after stroke: Systematic review and meta-analysis. Games Health J..

[27] Ghai S., Ghai I., Lamontagne A. (2020). Virtual reality training enhances gait poststroke: A systematic review and meta-analysis. Ann. N. Y. Acad. Sci..

[28] Xavier-Rocha T.B., Carneiro L., Martins G.C., Vilela-JÚnior G.B., Passos R.P., Pupe C.C.B., Nascimento O., Haikal D.S., Monteiro-Junior R.S. (2020). The Xbox/Kinect use in poststroke rehabilitation settings: A systematic review. Arq. Neuro-Psiquiatr..

[29] Lee H.S., Park Y.J., Park S.W. (2019). The Effects of virtual reality training on function in chronic stroke patients: A systematic review and meta-analysis. Biomed. Res. Int..

[30] Mohammadi R., Semnani A.V., Mirmohammadkhani M., Grampurohit N. (2019). Effects of virtual reality compared to conventional therapy on balance poststroke: A systematic review and meta-analysis. J. Stroke Cerebrovasc. Dis..

[31] Ahn S., Hwang S. (2019). Virtual rehabilitation of upper extremity function and independence for stoke: A meta-analysis. J. Exerc. Rehabil..

[32] Aminov A., Rogers J.M., Middleton S., Caeyenberghs K., Wilson P.H. (2018). What do randomized controlled trials say about virtual rehabilitation in stroke? A systematic literature review and meta-analysis of upper-limb and cognitive outcomes. J. Neuroeng. Rehabil..

[33] Ferreira V., Carvas N., Artilheiro M.C., Pompeu J.E., Hassan S.A., Kasawara K.T. (2020). Interactive video gaming improves functional balance in poststroke individuals: Meta-analysis of randomized controlled trials. Eval. Health Prof..

[34] Gibbons E.M., Thomson A.N., de Noronha M., Joseph S. (2016). Are virtual reality technologies effective in improving lower limb outcomes for patients following stroke—A systematic review with meta-analysis. Top. Stroke Rehabil..

[35] Iruthayarajah J., McIntyre A., Cotoi A., Macaluso S., Teasell R. (2017). The use of virtual reality for balance among individuals with chronic stroke: A systematic review and meta-analysis. Top. Stroke Rehabil..

[36] Chen L., Lo W.L., Mao Y.R., Ding M.H., Lin Q., Li H., Zhao J.L., Xu Z.Q., Bian R.H., Huang D.F. (2016). Effect of virtual reality on postural and balance control in patients with stroke: A systematic literature review. Biomed. Res. Int..

[37] Luque-Moreno C., Ferragut-Garcías A., Rodríguez-Blanco C., Heredia-Rizo A.M., Oliva-Pascual-Vaca J., Kiper P., Oliva-Pascual-Vaca Á. (2015). A decade of progress using virtual reality for poststroke lower extremity rehabilitation: Systematic review of the intervention methods. Biomed. Res. Int..

[38] Corbetta D., Imeri F., Gatti R. (2015). Rehabilitation that incorporates virtual reality is more effective than standard rehabilitation for improving walking speed, balance and mobility after stroke: A systematic review. J. Physiother..

[39] Li Z., Han X.G., Sheng J., Ma S.J. (2016). Virtual reality for improving balance in patients after stroke: A systematic review and meta-analysis. Clin. Rehabil..

[40] Rodrigues-Baroni J.M., Nascimento L.R., Ada L., Teixeira-Salmela L.F. (2014). Walking training associated with virtual reality-based training increases walking speed of individuals with chronic stroke: Systematic review with meta-analysis. Braz. J. Phys. Ther..

[41] Lohse K.R., Hilderman C.G., Cheung K.L., Tatla S., Van der Loos H.F. (2014). Virtual reality therapy for adults post-stroke: A systematic review and meta-analysis exploring virtual environments and commercial games in therapy. PLoS ONE.

[42] Laver K., George S., Ratcliffe J., Crotty M. (2011). Virtual reality stroke rehabilitation—Hype or hope?. Aust. Occup. Ther. J..

[43] Fu M.J., Knutson J.S., Chae J. (2015). Stroke rehabilitation using virtual environments. Phys. Med. Rehabil. Clin. N. Am..

[44] Lee H.C., Huang C.L., Ho S.H., Sung W.H. (2017). The effect of a virtual reality game intervention on balance for patients with stroke: A randomized controlled trial. Games Health J..

[45] Daly J.J., Ruff R.L. (2007). Construction of efficacious gait and upper limb functional interventions based on brain plasticity evidence and model-based measures for stroke patients. Sci. World J..

[46] Kim S.H., Cho S.H. (2022). Benefits of virtual reality program and motor imagery training on balance and fall efficacy in isolated older adults: A randomized controlled trial. Medicina.

[47] Holden M.K. (2005). Virtual environments for motor rehabilitation: Review. Cyberpsychol. Behav..

[48] Party I.S.W. (2012). National Clinical Guideline for Stroke.

[49] Schuster-Amft C., Eng K., Suica Z., Thaler I., Signer S., Lehmann I., Schmid L., McCaskey M.A., Hawkins M., Verra M.L. (2018). Effect of a four-week virtual reality-based training versus conventional therapy on upper limb motor function after stroke: A multicenter parallel group randomized trial. PLoS ONE.

[50] Moore J.L., Nordvik J.E., Erichsen A., Rosseland I., Bø E., Hornby T.G. (2020). Implementation of high-intensity stepping training during inpatient stroke rehabilitation improves functional outcomes. Stroke.

[51] Mekbib D.B., Zhao Z., Wang J., Xu B., Zhang L., Cheng R., Fang S., Shao Y., Yang W., Han J. (2020). Proactive motor functional recovery following immersive virtual reality-based limb mirroring therapy in patients with subacute stroke. Neurotherapeutics.

[52] Yoon H.J., Kim J., Park S.W., Heo H. (2020). Influence of virtual reality on visual parameters: Immersive versus non-immersive mode. BMC Ophthalmol..

